# Renal Transplantation in HIV-positive and HIV-negative People With Advanced Stages of Kidney Disease: Equity in Transplantation

**DOI:** 10.1093/ofid/ofae182

**Published:** 2024-04-16

**Authors:** Seyed M Hosseini-Moghaddam, Yuguang Kang, Sarah E Bota, Matthew A Weir

**Affiliations:** ICES, Kidney, Dialysis & Transplantation Research Program, London, Ontario, Canada; Multiorgan Transplant Program, University Health Network, University of Toronto, Toronto, Ontario, Canada; Division of Infectious Diseases, Department of Medicine, Western University, London, Ontario, Canada; ICES, Kidney, Dialysis & Transplantation Research Program, London, Ontario, Canada; Lawson Health Research Institute, London Health Sciences Centre, London, Ontario, Canada; ICES, Kidney, Dialysis & Transplantation Research Program, London, Ontario, Canada; Lawson Health Research Institute, London Health Sciences Centre, London, Ontario, Canada; ICES, Kidney, Dialysis & Transplantation Research Program, London, Ontario, Canada; Division of Nephrology, Department of Medicine, Western University, London, Ontario, Canada; Department of Epidemiology and Biostatistics, Western University, London, Ontario, Canada

**Keywords:** death, end stage kidney disease, graft failure, HIV infection, kidney transplantation

## Abstract

**Background:**

People with HIV are at a greater risk of end-stage kidney disease than the general population. Considering the risk of death after end-stage kidney disease, access to renal transplantation in people with HIV is critically important.

**Methods:**

We included all adult patients on chronic dialysis in Ontario, Canada, between 1 April 2007 and 31 December 2020. We determined the probability of kidney transplantation with competing risk of death over time since the initiation of dialysis by calculating the adjusted subdistribution hazard ratios (sdHR; 95% confidence interval [CI]). We also compared long-term renal allograft and posttransplant mortality outcomes between HIV-negative and HIV-positive persons.

**Results:**

Of 40 686 people (median age, 68 years; interquartile range, 57–77; 38.4% women), 173 were HIV-positive and 40 513 were HIV-negative. The incidence of kidney transplantation in HIV-negative and HIV-positive patients was 40.5 (95% CI, 39.4-41.6)/1000 person-years and 35.0 (95% CI, 22.8-53.7)/1000 person-years, respectively (*P* = .51). Considering the competing risk of death, HIV-positive people had a significantly lower chance of receiving kidney transplants than HIV-negative people (sdHR, 0.46 [95% CI, .30–.70]). The long-term allograft failure risk was not significantly different between HIV-negative and HIV-positive people, considering the competing risk of posttransplant death (sdHR, 1.71 [95% CI, .46-6.35]).

**Conclusions:**

Although the incidence and crude probability of kidney transplantation were similar among HIV-negative and HIV-positive persons in this cohort, those with HIV had a significantly lower likelihood of kidney transplantation than those without HIV. Having HIV was not significantly associated with a poor long-term allograft outcome compared with patients without HIV.

## Teaser

In this population-based study in Ontario, the likelihood of access to kidney transplantation in HIV-positive persons was significantly lower than in HIV-negative individuals, considering the competing risk of death. The long-term allograft outcome was not significantly affected by having HIV.

## Graphical Abstract

**Graphical Abstract:**
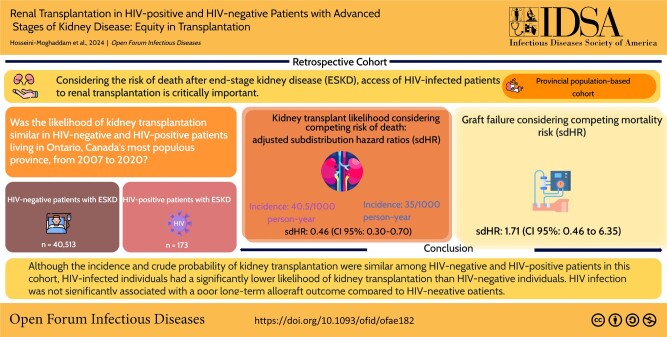
This graphical abstract is also available at Tidbit: https://tidbitapp.io/tidbits/renal-transplantation-in-hiv-positive-and-hiv-negative-patients-with-advanced-stages-of-kidney-disease-equity-in-transplantation

Chronic kidney disease incidence and prevalence are greater in those with HIV than in the general population [1, 2]. Those with HIV are at increased risk of end-stage kidney disease (ESKD) [3]. A nationwide, population-based study from Denmark showed an incidence rate ratio of 3.6 for ESKD requiring chronic dialysis when comparing a matched population of those with and without HIV [4]. Following the increasing accessibility of antiretroviral therapy (ART), the incidence of ESKD resulting from HIV-associated nephropathy (HIVAN) and HIVAN-associated mortality substantially decreased [5]. However, those with HIV and ESKD still experience substantial mortality, even in the current ART era [6]. The analysis of United States Renal Data Systems data for more than 1.5 decades demonstrated that despite improvement in 1-year survival of those with HIV and on dialysis, the overall outcome was inferior to the matched HIV-negative cohort [7].

Kidney transplantation typically confers a considerable survival benefit in people with HIV and ESKD [8]. Although kidney transplantation significantly improves the quality of life of patients with ESKD, those with HIV may have suboptimal access to renal transplantation compared with HIV-negative patients with ESKD [2]. Previous data from New York City from 2000 to 2007 showed that only 20% of those with HIV and 73% of those without HIV with ESKD achieved listing for kidney transplantation. Similarly, another study from Philadelphia showed despite the improvement in the wait-listing rate in those with HIV, the evaluation process was considerably longer for candidates with HIV compared with those without. Although access to transplantation is critically important in people with HIV, some transplant centers may be reluctant to offer this procedure to those with HIV because of the risk of HIVAN relapse, rejection, and drug-drug interaction [9]. Most studies comparing the accessibility to renal transplantation between HIV-positive and HIV-negative people did not consider the simultaneous risk of pretransplant death [10]. Calculating renal transplant incidence without considering the concurrent risk of death and the role of comorbidities may not accurately estimate the probability of transplantation [11]. Large-scale data are needed to compare the likelihood of renal transplantation between those with and those without HIV while considering the competing risk of mortality. The objectives of this study were to compare the incidence of kidney transplantation between HIV-positive and HIV-negative people with ESKD. We also compared the mortality and renal allograft failure between those with and without HIV after renal transplantation.

## METHODS

### Study Design and Setting

We conducted this population-based study in Ontario, Canada's, most populous province (14.7 million in 2020). Approximately half of Canadian organ transplant procedures are performed in Ontario [12]. We used the provincial health care administrative databases held at ICES (www.ices.on.ca), an independent, not-for-profit research organization. The use of the data in this project is authorized under section 45 of Ontario's Personal Health Information Protection Act and does not require review by a Research Ethics Board. The provincial government of Ontario provides a universal, publicly funded, single-payer health insurance system to residents. Under Ontario's health information privacy law, we could use the health care and demographic databases held at ICES without consent. This project's data access and use were authorized under section 45 of Ontario's Personal Health Information Protection Act, which does not require Research Ethics Board approval.

We reported the study findings in accordance with the REporting of studies Conducted using Observational Routinely-collected Data guideline and supporting checklist (Supplementary Table 1) [13].

### Data Sources

We used the Canadian Organ Replacement Register to ascertain a cohort of patients on chronic dialysis in the accrual period of this cohort. We subsequently used the Registered Persons Database for demographic information and vital status. Information from multiple other databases, such as the Ontario Health Insurance Plan, Same Day Surgery, and Ontario HIV database were linked at the individual level using a unique identifier (ICES key number) for each person in Ontario to derive baseline characteristics and covariates as presented in Supplementary Table 2.

### Study Population

**Figure 1.**
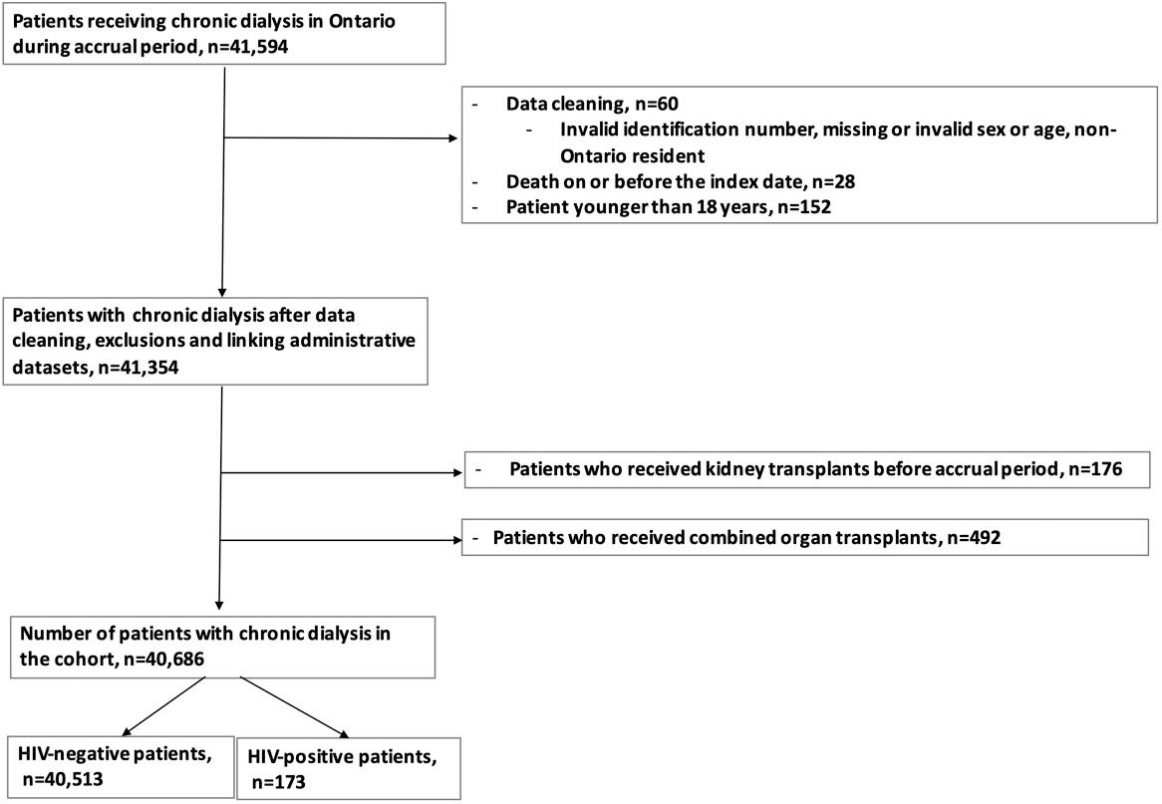
Study flow diagram. Patients on chronic dialysis and no contraindication to renal transplantation.

We created a cohort of adult patients with ESKD on chronic dialysis in Ontario, Canada, between 1 April 2007 and 31 December 2020, with a maximum follow-up date of 31 December 2021. After standard data cleaning (invalid or missing ICES key number, age, or sex, death on or before the index, or non-Ontario residents), we excluded patients aged 18 years or younger, those with a date of kidney transplant before the accrual period, and patients who received combined organ transplants (study flow chart, Figure 1).

### Patient Characteristics

We considered the first treatment date for chronic dialysis in the accrual period as the index date. Baseline characteristics included demographic data, neighborhood income quintiles, dialysis in the Greater Toronto Area (GTA) consisting of the city of Toronto and 4 surrounding regional municipalities (Durham, Halton, Peel, and York) versus non-GTA, living in rural versus urban areas, dialysis modality and comorbidities (ie, diabetes mellitus, chronic obstructive pulmonary disease [COPD], chronic lung disease, hypertension, cirrhosis, heart failure, major cancer, vascular disease, stroke/transient ischemic attack, and Deyo-Charlson Comorbidity Index) [14]. The exposure was that of having HIV using the Ontario HIV Database [15].

### Study Outcomes

The primary outcome was kidney transplantation. The secondary outcomes included pretransplant all-cause death, posttransplant death, and renal allograft failure. The end date of the observation period was 31 December 2021.

### Statistical Analysis

We determined baseline characteristics at the time of index date as frequencies (and percentages) for categorical variables and median (interquartile ranges [IQR]) for continuous variables. In accordance with ICES privacy policies, cell sizes ≤5 cannot be reported. We used standardized differences for comparison between exposed (ie, those with HIV) and unexposed (HIV-negative people) groups where a difference ≥0.10 is considered clinically meaningful. We compared the incidence rate of transplants per 1000 person-years in HIV-positive and HIV-negative people. We compared the access to kidney transplantation or outcome of death in the intervals of 2007–2012 and 2013–2020. We subsequently calculated the cumulative incidence of kidney transplant after dialysis initiation for HIV-positive and HIV-negative people. We determined the probability of kidney transplantation with the competing risk of death over time since the initiation of dialysis in the cohort by estimating the cumulative incidence function of kidney transplants in HIV-positive and HIV-negative people with pretransplant death considered as a competing risk of being transplanted using a Fine-Gray subdistribution hazard model. We measured the crude and adjusted subdistribution hazard ratios (sdHR, 95% confidence interval [CI]) accounting for the covariates in the adjusted models, including age, sex, income quintile, and city of dialysis (GTA vs non-GTA). We tested the proportional hazards assumption by assessing the weighted Schoenfeld residuals.

In a set of sensitivity analyses, we performed the same statistical tests on a subcohort of patients with ESKD who had no recorded contraindications to kidney transplant. In these analyses, we restricted the cohort to individuals with ESKD and without any of those 7 characteristics shown by Wang et al. are not present in >97% of patients receiving renal transplant: ESKD-modified Charlson Comorbidity Index score ≥7, home oxygen therapy, age >75 years, dementia, staying in a long-term care center, receiving ≥1 physician house visit in the past 365 days, a combination of select malignancies (ie, lymphoma, active multiple myeloma, lung, cervical, bladder, liver, and colorectal cancer) [16].

Next, we restricted the cohort to kidney transplant recipients. We used the Fine-Gray subdistribution hazard model to compare the outcome of allograft failure (ie, retransplantation or return to chronic dialysis) and Cox proportional hazards regression models to compare the risk of posttransplant death between renal allograft recipients with and without HIV. In the analysis of allograft failure, death was considered a competing risk event. Statistical tests were 2-sided, with the level of significance set at α = 0.05. All analyses were performed at ICES using SAS version 9.4 (SAS Institute, Cary, NC).

## RESULTS

### Patient Characteristics

We identified 41 594 patients who received maintenance dialysis treatment in Ontario during the accrual period of this cohort. After exclusion, 40 686 patients (median age, 68 years; IQR, 57-77; 38.4% women) including 173 people with HIV and 40 513 those without HIV. People with HIV were significantly younger than HIV-negative individuals (median [IQR]: 53 [46-61] versus 68 [57-77] years; standard deviation [SD], 0.35). The frequency of female patients with HIV was significantly lower than those without HIV (39/173 [22.5%] versus 15 581/40 513 [38.5%]; SD, 1.01]. Those with HIV had a significantly lower frequency of cancer (19/173 [11%] versus 6483/40 513 [16%]; SD, 0.15), COPD (29/173 [16.8%] versus 10 877/40 513 [26.8%]; SD, 0.24), diabetes mellitus (78/173 [45.1%] versus 24 820/40 513 [61.3%]; SD, 0.33), hypertension (117/173 [67.6%] versus 36 013/40 513 [88.9%]; SD, 0.53), heart failure (35/173 [20.2%] versus 14 335/40 513 [35.4%]; SD, 0.34) compared with HIV-negative individuals. Table 1 provides the comparison of demographic variables and comorbidities between HIV-positive and HIV-negative individuals.

**Table 1.** Baseline Characteristics of Patients With End-stage Kidney Disease by HIV Status

Characteristics	HIV Positive (n = 173), N (%)	HIV Negative (n = 40 513), N (%)	Standardized Difference
Index year			
2007-2012	53 (30.6)	13 448 (33.2)	.06
2013-2020	120 (69.4)	27 065 (66.8)	.06
Age, median (IQR)	53 (46–61)	68 (57-77)	1.01
Sex, female	39 (22.5)	15 581(38.5)	**.35**
Neighborhood income quintile			
1 (Lowest)	79 (45.7)	10 702(26.4)	**.41**
2	32 (18.5)	8992 (22.2)	.09
3	26 (15)	7743 (19.1)	**.11**
4	19 (11)	6949 (17.2)	**.18**
5	16 (9.2)	5940 (14.7)	**.17**
Rurality	6 (3.5)	4871 (12)	.**32**
City of dialysis, resides in the Greater Toronto Area (GTA)	104 (60.1)	16 628 (41)	.**39**
Dialysis modality			
HD	151 (87.3)	32 623 (80.5)	**.19**
PD	22 (12.7)	7882 (19.5)	**.19**
Combined	0	8 (0.02)	**.19**
Cancer	19 (11)	6483 (16)	.**15**
COPD	29 (16.8)	10 877 (26.8)	.**24**
Chronic lung disease	64 (37)	15 447 (38.1)	.02
Cirrhosis	9 (5.2)	1997 (4.9)	.01
Diabetes mellitus	78 (45.1)	24 820 (61.3)	.**33**
Hypertension	117 (67.6)	36 013 (88.9)	.**53**
Heart failure	35 (20.2)	14 335 (35.4)	.**34**
Vascular disease	11 (6.4)	2632 (6.5)	.00
Stroke/TIA	9 (5.2)	1951 (4.8)	.02
Charlson comorbidity index			
Median (IQR)	4 (2-9)	4 (2-5)	**.34**
2	61 (35.3)	16 051 (39.6)	.09
3	14 (8.1)	3502 (8.6)	.02
4	18 (10.4)	7412 (18.3)	**.23**
≥5	80 (46.2)	13 548 (33.4)	**.26**

We used bold font for standardized differences of comparison between HIV-positive and HIV-negative patients where a difference ≥0.10 is considered clinically meaningful.

Abbreviations: COPD, chronic obstructive pulmonary disease; GTA, The Greater Toronto Area including the city of Toronto and regional municipalities of Durham, Halton, Peel, and York; HD, hemodialysis; PD, peritoneal dialysis; TIA, transient ischemic attack.

### Kidney Transplantation

By the end of follow-up, 13.1% of HIV-negative (5313/40 513) and 12.1% of HIV-positive (21/173) people received kidney transplants in this cohort (*P* = .70) (Table 2). The rate of pretransplant death in HIV-negative (23 432/40 513) and HIV-positive people (90/173) was not significantly different (57.8% vs 52.0%, *P* = .12). The incidence of kidney transplantation in those with and without HIV was 40.5 (95% CI, 39.4-41.6)/1000 person-years) and 35.0 (95% CI, 22.8-53.7)/1000 person-years, respectively. The comparison of kidney transplantation incidence between those with and without HIV in the 2007 through 2012 interval (44.2 [95% CI, 42.5-45.9] versus 41.0 [95% CI, 21.4-78.9]/1000 person-years, respectively; *P* = .77) and the interval of 2013 through 2020 (37.7 [95% CI, 36.3-39.1] versus 31.5 [95% CI, 17.9-55.5]/1000 person-years, respectively, *P* = .89) did not demonstrate statistically significant difference.

**Table 2.** Outcomes of Kidney Transplantation and pretransplant Death in HIV-positive and HIV-negative Patients

Outcomes	HIV-positiveN = 173	HIV-negativeN = 40 513	Standardized Difference
Kidney transplantation, N (%)	21 (12.1%)	5313 (13.1%)	0.03
Pretransplant death, N (%)	90 (52.0%)	23 432 (57.8%)	0.12
Allograft failure	<6	19 (0.4%)	0.28-0.77*
Posttransplant death	<6	861 (16.2%)	0.05-0.38*

*The standardized difference reported as a range based on the possible values of 1–5.

#### Patients Without Contraindications to Kidney Transplantation

In sensitivity analyses, we limited the study population to a subcohort of 26 017 patients without contraindications to kidney transplantation (Figure 1). Of these, 25 868 were HIV-negative (99.4%) and 149 had HIV (0.6%). Overall, 5194/25 868 HIV-negative people (20.1%) and 20/149 HIV-positive people (13.4%) received kidney transplant (*P* = .04). The rate of pretransplant death in HIV-negative (11 840/25 868) and HIV-positive people (75/149) was not significantly different (45.8% vs 50.3%, *P* = .26). The incidence of kidney transplantation in HIV-negative and HIV positive people was 55.4 (95% CI, 53.9-56.9)/1000 person-years and 36.9 (95% CI, 23.8-57.2)/1000 person-years, respectively (*P* = 0. .07).

#### Likelihood of Kidney Transplantation

**Figure 2.**
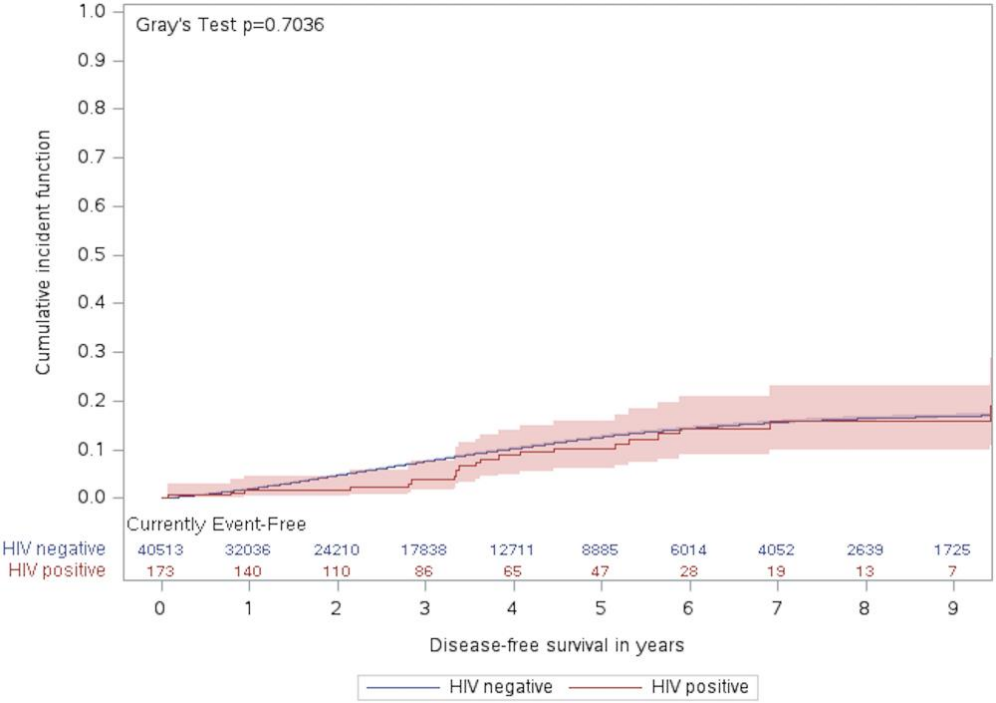
Cumulative incidence of kidney transplant during the follow-up period by HIV status.

The likelihood of kidney transplantation, considering the competing risk of death, was not significantly different between those with and without HIV in the crude sdHR (0.91 [95% CI, .60-1.39]). However, after adjusting for potential confounders including age, sex, income quintile, and city of dialysis (GTA vs non-GTA), those with HIV had a significantly lower chance of receiving kidney transplants than those without HIV (sdHR, 0.46 [95% CI, .30-.70]; *P* < .001). Figure 2 demonstrates the cumulative incidence function of kidney transplant during the follow-up period in HIV-positive and HIV-negative people.

#### Kidney Transplantation Outcome

Overall, 5334 patients in this cohort received kidney transplant. Of these, 19 without HIV (0.4%) and <6 with HIV developed allograft failure during follow-up period (sdHR, 1.48 [95% CI, .39-5.56]; *P* = .57). Similarly, allograft failure was no significant different between HIV-positive and HIV-negative people in the model adjusted for city of dialysis (GTA vs non-GTA), age, and sex (sdHR, 1.71 [95% CI, .46-6.35]; *P* = .43). Overall, 861/5313 HIV-negative people (16.2%), and <6 HIV-positive people died during posttransplant observation period (HR, 0.35 [95% CI, .05-2.44]; *P* = .2) (Table 2). Likewise, in the model adjusted for city of dialysis (GTA vs non-GTA), age, and sex, the outcome of posttransplant mortality was not significantly different between those with and without HIV (HR, 0.63 [95% CI, .09-4.49]; *P* = .65).

## DISCUSSION

In this large-scale, population-based 14-year cohort, we estimated the incidence of kidney transplantation in Ontario, a large province with approximately 40% of Canada's population [17]. We showed the likelihood of kidney transplantation in those with HIV was significantly lower than in HIV-negative individuals in the entire cohort. In crude models, the cumulative incidence of kidney transplantation and pretransplant mortality in those with and without HIV was not significantly different. The kidney transplant incidence rate was not significantly different between HIV-positive and HIV-negative people in 2 intervals of 2007 through 2012 and 2013 through 2020. In sensitivity analysis, when we limited the crude analysis to a subgroup of individuals without contraindication to kidney transplantation procedure, we observed a lower proportion of kidney transplantation in HIV-positive individuals compared with HIV-negative people (13.4% vs 20.1%, *P* = .04), whereas the pretransplant death was relatively similar in these 2 subgroups (45.8% in those without HIV and 50.3% in those with HIV; *P* = .24). In this subcohort, the incidence of kidney transplantation in HIV-negative people was greater than HIV-positive individuals (55.4/1000 person-years vs 36.9/1000 person-years, respectively; *P* = .07). These crude analyses did not uncover a suboptimal access to kidney transplantation in people with HIV with ESKD. However, the adjusted Fine-Gray subdistribution hazard model showed a significantly lower access to kidney transplantation in HIV-positive people than HIV-negative individuals with ESKD.

Although health care and dialysis were provided at no cost for those with HIV in Ontario, the likelihood of kidney transplantation in people with HIV and ESKD was considerably lower than in HIV-negative individuals with ESKD. The reasons for this issue do not appear to be related to comorbidities because HIV-positive persons had a significantly lower frequency of cancer, COPD, diabetes mellitus, hypertension, and heart failure compared with HIV-negative persons. Similarly, a lower likelihood of renal transplantation in HIV-positive people does not seem to be related to geographic access because the frequencies of living in urban (vs rural) areas and GTA (vs non-GTA) were significantly greater in HIV-positive people than in HIV-negative people. The potential explanations may include the medical conditions associated with HIV, socioeconomic factors, and probably reluctance of transplant programs and health care providers because of complex drug-drug interactions between immunosuppressive agents and ART regimens, risk of HIVAN relapse, and allograft rejection. The advantages of kidney transplantation in those with HIV and the existing inequity of access to kidney transplantation should be highlighted for transplant programs. In the analysis of United States Renal Data Systems data of more than a decade, improvement in 1-year survival was observed in people with HIV and ESKD following renal transplantation. However, despite improved access to this procedure in the United States, there are still substantial barriers to kidney transplantation referrals in people with HIV [18]. Similarly, new studies from the central and east Europe network, including 19 countries, demonstrated that people with HIV have significantly limited access to kidney transplantation despite full access to kidney disease screening [19]. Further observational studies and surveillance programs are required to monitor the improvement of disparities in access to kidney transplantation in HIV-positive and HIV-negative persons.

Kidney transplantation was associated with poor allograft outcomes and patient survival before the introduction of potent ART [20]. However, kidney transplantation in the ART era was associated with 69% allograft survival at a median follow-up of 4 years, as reported by the Scientific Registry of Transplant Recipients [21]. Although the long-term outcome of kidney transplantation in people with HIV is still unclear, propensity-matched analysis of up to 15-year follow-ups, including 119 HIV-positive and 655 HIV-negative people, demonstrated that having HIV is not associated with worse long-term renal allograft survival [22]. In our cohort, the risk of allograft failure and posttransplant death was not significantly different between HIV-positive and HIV-negative people.

The strength of our study lies in its population-based design. We provided the long-term outcome of kidney transplantation comparing HIV-positive and HIV-negative people. In a retrospective study of the United Network for Organ Sharing database, an increased risk of mortality and allograft failure was observed in people with both HIV and hepatitis C virus. However, there was no significant difference in allograft or patient survival between HIV-monoinfected and HIV-negative individuals [23]. Our study was one of the largest cohorts of patients with ESKD, with accrual periods extending from 2007 to 2021. One of the study's strengths is the concordance of the findings of the analyses in the entire cohort and a subcohort of patients with no contraindication to kidney transplant in sensitivity analyses.

Our study was also associated with some limitations. First, we did not have access to the waiting list of individual transplant centers across the province; therefore, we could not compare the likelihood of being listed for transplantation between HIV-positive and HIV-negative people with ESKD. Different transplant programs did not necessarily have similar approaches for waitlisting. Instead, we used a validated method to identify individuals with contraindications to kidney transplantation in a sensitivity analysis. Second, we did not have data to determine the frequency of posttransplant events at the individual level, such as allograft rejection. The secondary endpoint of interest in this cohort was graft loss and not allograft rejection episodes. Third, although we observed no significant difference in long-term allograft outcomes between HIV-positive and HIV-negative people, this finding requires cautious interpretation considering the small number of HIV-positive people with allograft failure or posttransplant death (n < 6).

## CONCLUSION

This population-based cohort demonstrated a significant difference between HIV-positive and HIV-negative people with ESKD in kidney transplantation. Considering the substantial risk of pretransplant death and improvement of survival following kidney transplantation, all efforts should be made to reduce this disparity. Our study, with more than 14 years of observation, did not show a significant difference in allograft survival comparing HIV-positive and HIV-negative kidney transplant patients. We recommend monitoring and surveillance of access to kidney transplantation in HIV-positive people with ESKD to reduce the observed gap. We also recommend further studies to capture potential reasons for the differences in the frequency of kidney transplantation between HIV-positive and HIV-negative people.

## Supplementary Material

ofae182_Supplementary_Data

## Supplementary Data

Supplementary materials are available at *Open Forum Infectious Diseases* online. Consisting of data provided by the authors to benefit the reader, the posted materials are not copyedited and are the sole responsibility of the authors, so questions or comments should be addressed to the corresponding author.

## Notes

***Acknowledgments.*** This study was supported by ICES, which is funded by an annual grant from the Ontario Ministry of Health (MOH) and the Ministry of Long-Term Care (MLTC). The study was completed at the ICES Western site. This study also received funding from Department of Medicine at Western University. This document used data adapted from the Statistics Canada Postal Code^OM^ Conversion File, which is based on data licensed from Canada Post Corporation, and/or data adapted from the Ontario Ministry of Health Postal Code Conversion File, which contains data copied under license from Canada Post Corporation and Statistics Canada. We thank IQVIA Solutions Canada Inc. for use of their Drug Information File. Parts of this material are based on data compiled and provided by the Ontario Ministry of Health, the Canadian Institute for Health Information, and Ontario Health (OH). The analyses, conclusions, opinions, and statements expressed herein are solely those of the authors and do not reflect those of the funding or data sources; no endorsement is intended or should be inferred.

***Author Contributions.* S.M.H.:** study concept and design, data interpretation, drafting of the manuscript; **Y.K.:** analysis and interpretation of data, critical revision of the manuscript; **S.E.B.:** study design, data analysis and interpretation, critical revision of the manuscript; **M.A.W.:** study concept and design, critical revision of the manuscript.

***Disclaimer.*** No author has potential financial, professional, or personal conflicts relevant to the manuscript.

***Patient consent statement.*** The use of the data in this project is authorized under section 45 of Ontario's Personal Health Information Protection Act (PHIPA) and does not require review by a Research Ethics Board. The provincial government of Ontario provides a universal, publicly funded, single-payer health insurance system to residents. Under Ontario's health information privacy law, we could use the healthcare and demographic databases held at ICES without consent. This project's data access and use were authorized under section 45 of Ontario's Personal Health Information Protection Act, which does not require Research Ethics Board approval.

***Financial support*.** This study was supported by Multi-Organ Transplant Program (MOTP) at London Health Sciences Centre (LHSC) in London, Ontario, Canada, Western University. The analyses, conclusions, opinions, and statements expressed herein are solely those of the authors and do not reflect those of the funding or data sources; no endorsement is intended or inferred.
