# Supplementary material for: Renal Transplantation in HIV-positive and HIV-negative People With Advanced Stages of Kidney Disease: Equity in Transplantation
Source: Open Forum Infect Dis. 2024 Apr 16;11(5):ofae182. doi: 10.1093/ofid/ofae182 (PMC11083635; doi:10.1093/ofid/ofae182)
Supplement: ofae182_Supplementary_Data [file ofae182_supplementary_data.docx]

# Appendix- Access to Renal Transplantation Considering HIV Serostatus of Patients with Advanced Stages of Renal Failure: Equity in Transplantation

**Supplemental Table 1.** Checklist of REporting of studies Conducted using Observational Routinely-collected health Data (RECORD) Statement

|  | Item No | STROBE items | RECORD items | Reported |
| --- | --- | --- | --- | --- |
| Title and abstract | 1 | (a) Indicate the study's design with a commonly used term in the title or the abstract.  (b) Provide in the abstract an informative and balanced summary of what was done and what was found. | (1.1) The type of data used should be specified in the title or abstract. When possible, the name of the databases used should be included.  (1.2) If applicable, the geographic region and time frame within which the study took place should be reported in the title or abstract.  (1.3) If linkage between databases was conducted for the study, this should be clearly stated in the title or abstract. | Abstract |
| Introduction |  |  |  |  |
| Background/ rationale | 2 | Explain the scientific background and rationale for the investigation being reported. |  | Introduction |
| Objectives | 3 | State specific objectives, including any prespecified hypotheses. |  | Introduction |
| Methods |  |  |  |  |
| Study design | 4 | Present key elements of study design early in the paper. |  | Methods |
| Setting | 5 | Describe the setting, locations, and relevant dates, including periods of recruitment, exposure, follow-up, and data collection. |  | Methods |
| Participants | 6 | (a) Give the eligibility criteria, and the sources and methods of selection of participants. Describe methods of follow-up.  (b) For matched studies, give matching criteria and number of exposed and unexposed. | (6.1) The methods of study population selection (such as codes or algorithms used to identify subjects) should be listed in detail. If this is not possible, an explanation should be provided.  (6.2) Any validation studies of the codes or algorithms used to select the population should be referenced. If validation was conducted for this study and not published elsewhere, detailed methods and results should be provided.  (6.3) If the study involved linkage of databases, consider use of a flow diagram or other graphical display to demonstrate the data linkage process, including the number of individuals with linked data at each stage. | Methods,  Appendix |
| Variables | 7 | Clearly define all outcomes, exposures, predictors, potential confounders, and effect modifiers. Give diagnostic criteria, if applicable. | (7.1) A complete list of codes and algorithms used to classify exposures, outcomes, confounders, and effect modifiers should be provided. If these cannot be reported, an explanation should be provided. | Methods, Appendix |
| Data sources/   measurement | 8 | For each variable of interest, give sources of data and details of methods of assessment (measurement). Describe comparability of assessment methods if there is more than one group. |  | Methods, Appendix |
| Bias | 9 | Describe any efforts to address potential sources of bias. |  | Methods, Discussion |
| Study size | 10 | Explain how the study size was arrived at. |  | Methods, Results |
| Quantitative variables | 11 | Explain how quantitative variables were handled in the analyses. If applicable, describe which groupings were chosen and why. |  | Methods |
| Statistical methods | 12 | (a) Describe all statistical methods, including those used to control for confounding.  (b) Describe any methods used to examine subgroups and interactions.  (c) Explain how missing data were addressed.  (d) If applicable, explain how loss to follow-up was addressed.  (e) Describe any sensitivity analyses. |  | Methods |
| Data access and cleaning methods |  | N/A | (12.1) Authors should describe the extent to which the investigators had access to the database population used to create the study population.  (12.2) Authors should provide information on the data cleaning methods used in the study. | Methods |
| Linkage |  | N/A | (12.3) State whether the study included person-level, institutional-level, or other data linkage across two or more databases. The methods of linkage and methods of linkage quality evaluation should be provided. | Methods |
| Results |  |  |  |  |
| Participants | 13 | (a) Report numbers of individuals at each stage of study--e.g. numbers potentially eligible, examined for eligibility, confirmed eligible, included in the study, completing follow-up, and analyzed.  (b) Give reasons for non-participation at each stage.  (c) Consider use of a flow diagram. | (13.1) Describe in detail the selection of the persons included in the study (i.e., study population selection), including filtering based on data quality, data availability, and linkage. The selection of included persons can be described in the text and/or by means of the study flow diagram. | Methods, Results |
| Descriptive data | 14 | (a) Give characteristics of study participants (e.g. demographic, clinical, social) and information on exposures and potential confounders.  (b) Indicate number of participants with missing data for each variable of interest.  (c) Summarize follow-up time (e.g. average and total amount). |  | Results |
| Outcome data | 15 | Report numbers of outcome events or summary measures over time. |  | Results |
| Main results | 16 | (a) Give unadjusted estimates and, if applicable, confounder-adjusted estimates and their precision (e.g. 95% confidence interval). Make clear which confounders were adjusted for and why they were included.  (b) Report category boundaries when continuous variables were categorized.  (c) If relevant, consider translating estimates of relative risk into absolute risk for a meaningful time period. |  | Results |
| Other analyses | 17 | Report other analyses done (e.g. analyses of subgroups and interactions, and sensitivity analyses). |  | Results |
| Key results | 18 | Summarize key results with reference to study objectives. |  | Discussion |
| Limitations | 19 | Discuss limitations of the study, taking into account sources of potential bias or imprecision. Discuss both direction and magnitude of any potential bias. | (19.1) Discuss the implications of using data that were not created or collected to answer the specific research question(s). Include discussion of misclassification bias, unmeasured confounding, missing data, and changing eligibility over time, as they pertain to the study being reported. | Discussion |
| Interpretation | 20 | Give a cautious overall interpretation of results considering objectives, limitations, multiplicity of analyses, results from similar studies, and other relevant evidence. |  | Discussion |
| Generalizability | 21 | Discuss the generalizability (external validity) of the study results. |  | Discussion |
| Other information | |  |  |  |
| Funding | 22 | Give the source of funding and the role of the funders for the present study and, if applicable, for the original study on which the present article is based. |  | Acknowledgments & Funding |
| Accessibility of protocol, raw data, and programming code |  | N/A | (22.1) Authors should provide information on how to access any supplemental information such as the study protocol, raw data, or programming code. | Methods,  Appendix |

**Supplement Table 2.** Data Sources Used in the Study

| Source | Full Name | Description |
| --- | --- | --- |
| CIHI DAD | Canadian Institute for Health Information-Discharge Abstract Database | The CIHI DAD database contains summarized hospital discharge information for individuals receiving inpatient care in a non-mental health designated bed. Each record contains the patient identifier, the date of admission and discharge, clinical details of the hospitalization (including diagnoses made and procedures received). |
| OHIP | Ontario Health Insurance Plan Claims Database | The OHIP database contains Ontario physician claims from inpatient, outpatient, and long-term care settings. Each record identifies the physician, the patient, the diagnosis responsible for the claim (which follows the coding scheme in the International Classification of Diseases, 9th edition), the service provided, and the date on which the service was provided. |
| ODB | Ontario Drug Benefit | This database contains Ontario Drug Benefit (ODB) information, including recipients, payment, claims, and pharmacy and practitioner information. |
| SDS | Same Day Surgery Database | This database contains information related to surgical procedures do not require hospitalization |
| CIHI | Canadian Institute for Health Information | CIHI contains information related to medical or surgical interventions. |
| NACRS | National Ambulatory Care Reporting System | NACRS contains information on patient visits to hospital and community-based ambulatory and emergency care. Each record contains the patient identifier, date of registration, and clinical details of the visit. |
| RPDB | Registered Persons Database | The RPDB contains demographic information such as age, sex, health insurance eligibility and death information for anyone who has received Ontario health care coverage. It contains postal code information that is linkable to other geographic information such as dissemination areas which are needed to link to the Ontario Marginalization Index and other census-derived, neighborhood-level information. |
| LHIN | Local Health Integration Networks | Local Health Integration Network (LHIN) includes health related coding and geographic information in Ontario |
| INST | Health care institutions database | This databased includes information about Ontario health care institutions funded by the Ministry of Health and Long-Term Care (MOHLTC) |
| HIV | ICES-derived cohort | This databased includes information of patients living with HIV in Ontario |
| ADP | Assistive Devices Program | This database contains information related to Ontario Assistive Devices Program, e.g., Left ventricular assisting device |
| CONTACT | Population and demographics | This database includes all information of annual health services contacts in Ontario. |
| CORR | Canadian Organ Replacement Register | CORR is a pan-Canadian information system managed by CIHI. CORR contains data from all hospital dialysis programs, transplant programs, organ procurement organizations (OPOs) and independent health facilities to track patients from their first treatment for end stage organ failure including dialysis or organ transplantation to their death. |
| COPD | Ontario Chronic Obstructive Pulmonary Disease | The Ontario COPD cohort contains all Ontario COPD patients identified since 1991. |
| ODD | Ontario Diabetes Dataset | ICES dataset containing provincial data related to patients with diabetes mellitus |
| PCCF | Coding and geography database | This dataset is a postal code conversion file in Ontario |
| REF | Coding and geography database | This is a geographic/coding registry dataset in Ontario |
| Dementia | ICES-derived cohort | Ontario Dementia Database is a |
| Ontario Cancer registry | ICES-derived cohort | Ontario Cancer Registry includes data related to diagnosis and treatment of cancer in Ontario from 1964 to 2022. |
